# KLHL12 Promotes Non-Lysine Ubiquitination of the Dopamine Receptors D_4.2_ and D_4.4_, but Not of the ADHD-Associated D_4.7_ Variant

**DOI:** 10.1371/journal.pone.0145654

**Published:** 2015-12-30

**Authors:** Kamila Skieterska, Pieter Rondou, Béatrice Lintermans, Kathleen Van Craenenbroeck

**Affiliations:** Laboratory of GPCR Expression and Signal Transduction (L-GEST), Ghent University, Ghent, Belgium; University of California, Davis, UNITED STATES

## Abstract

**Dopamine D_4_ Receptor Polymorphism:**

The dopamine D_4_ receptor has an important polymorphism in its third intracellular loop that is intensively studied and has been associated with several abnormal conditions, among others, attention deficit hyperactivity disorder.

**KLHL12 Promotes Ubiquitination of the Dopamine D_4_ Receptor on Non-Lysine Residues:**

In previous studies we have shown that KLHL12, a BTB-Kelch protein, specifically interacts with the polymorphic repeats of the dopamine D_4_ receptor and enhances its ubiquitination, which, however, has no influence on receptor degradation. In this study we provide evidence that KLHL12 promotes ubiquitination of the dopamine D_4_ receptor on non-lysine residues. By using lysine-deficient receptor mutants and chemical approaches we concluded that ubiquitination on cysteine, serine and/or threonine is possible.

**Differential Ubiquitination of the Dopamine D_4_ Receptor Polymorphic Variants:**

Additionally, we show that the dopamine D_4.7_ receptor variant, which is associated with a predisposition to develop attention deficient hyperactivity disorder, is differentially ubiquitinated compared to the other common receptor variants D_4.2_ and D_4.4_. Together, our study suggests that GPCR ubiquitination is a complex and variable process.

## Introduction

Dopamine is an important neurotransmitter in the mammalian brain that controls many basic processes, such as reward-motivated behavior, emotion, movement, sexual behavior and endocrine regulation, by binding to the specific receptors at the postsynaptic and presynaptic terminal. Dopamine receptors (DR) belong to the superfamily of G protein-coupled receptors (GPCR) and are divided into two classes: dopamine D_1_-like receptors (including D_1_R and D_5_R) that bind to G_s_ proteins and activate adenylyl cyclase and dopamine D_2_-like receptors (including D_2_R, D_3_R and D_4_R) that inhibit adenylyl cyclase via G_i/o_ proteins [1–3].

The dopamine D_4_ receptor (D_4_R) has an important polymorphism in its third intracellular loop (IC3) where a 16-amino acid sequence is repeated 2 to 11 times resulting in different receptor variants referred to as D_4.2_R to D_4.11_R [4]. Although many variants exist, three of them are the most frequent in the human population, respectively D_4.4_R (64%), D_4.7_R (21%) and D_4.2_R (8%) [5]. The functional role of this polymorphism is not well characterized but several studies suggest association of the 7-repeat variant (D_4.7_R) with a predisposition to develop attention deficit hyperactivity disorder (ADHD) [6, 7]. In our previous study we have identified by yeast two-hybride screening a BTB-Kelch protein, KLHL12, which specifically interacts with the polymorphic repeats of the D_4_R and functions as adaptor in the Cullin3-based E3 ligase complex promoting ubiquitination of the receptor [8].

Ubiquitination is a posttranslational modification involving a covalent attachment of a 76-amino acid polypeptide, ubiquitin, predominantly to lysine residues in the substrate protein. Ubiquitin can be attached to a single lysine residue resulting in monoubiquitination or to multiple lysines in the substrate protein resulting in multi-monoubiquitination. Ubiquitin itself contains seven lysine residues and all of them can serve as ubiquitin binding sites. Addition of an extra ubiquitin to the previous substrate-bound ubiquitin molecule leads to the formation of polyubiquitin chains of different configuration [9, 10].

Unconventional ubiquitination is the phenomenon suggested for the first time by Berejtschopf et al. in 1998 [11] and refers to ubiquitin binding to non-lysine residues. Until now, besides on lysine, ubiquitination on the N-terminus of the polypeptide backbone [11–13], on cysteine [14–16], on serine and threonine [17–19] residues has been described. Different types of ubiquitination can lead to different functional effects. The best characterized role of ubiquitination is to mark its target proteins for proteasomal degradation, which is mainly mediated by lysine 48-linked polyubiquitination, and lysosomal degradation. Additionally, ubiquitination can also be involved in many other processes, such as protein trafficking, DNA repair, inflammation and protein translation [9, 20]. The unconventional ubiquitination seems to have similar functions as typical lysine-mediated ubiquitination and works mainly as a signal for protein degradation [14, 17–19, 21].

Another interesting feature of conventional and unconventional ubiquitination is their redundancy. When the most preferred ubiquitin binding site is not available, ubiquitin can be attached to another lysine or non-lysine residue in the substrate protein [17–19, 21]. Finally, ubiquitination is also a reversible process in which ubiquitin can be removed from the substrate protein by deubiquitinating enzymes.

Ubiquitination also plays an important role in GPCR regulation. Two major types of GPCR ubiquitination can be identified. The first one, agonist-dependent ubiquitination of plasma membrane receptors, functions as a sorting signal for lysosomal degradation. This mechanism is important for signal termination of receptors after prolonged agonist stimulation or irreversibly activated receptors [22]. In this case, stimulated receptors are targeted to lysosomes via the highly conserved endosomal-sorting complex required for transport (ESCRT) pathway [22–28].

The second major type of GPCR ubiquitination is the agonist-independent or constitutive ubiquitination. Basally ubiquitinated receptors can be present both at the plasma membrane and in the endoplasmic reticulum (ER). Ubiquitination of GPCRs during synthesis in the ER functions mainly as a quality control system in which misfolded receptors are ubiquitinated and targeted for degradation via the ER-associated degradation (ERAD) pathway [29]. However, there are examples described of properly folded GPCRs e.g. Adenosine receptor A_2A_ that are directed from the ER to the proteasome and after deubiquitination are transported to the cell surface [30].

We have investigated before the role of KLHL12-mediated ubiquitination in D_4_R degradation but no influence of KLHL12 on the expression level of D_4_R or on the half-life of the receptor were observed, allowing us to conclude that this specific ubiquitination does not lead to receptor degradation [31].

In this current work we investigate which specific residues in the sequence of the D_4_R are ubiquitinated and provide evidence for the ubiquitination on non-lysine residues. This poorly studied phenomenon has so far only once been suggested for a GPCR [32]. Moreover, we also show for the first time that the D_4.7_R variant, which is associated with a predisposition to develop ADHD, is differentially ubiquitinated compared to the other common receptor polymorphic variants, namely D_4.2_R and D_4.4_R, suggesting the possibility of different downstream signaling effects.

## Materials and Methods

### Plasmids and antibodies

Plasmids encoding HA D_4.2_R, HA D_4.4_R, HA D_4.7_R, HA D_4.0_R, Flag Ubiquitin, Etag KLHL12, Cmyc Cullin3, were described before [8]. The D_4.2_R contains only four lysine (K) residues in its whole primary structure, namely at sites 229 (in the third intracellular loop before the polymorphic region), 304, 311 (both in the third intracellular loop after the polymorphism) and 381 (in the C-terminal tail). The HA D_4.2_R construct was used as a template for the application of single-nucleotide mutations (encoding arginine instead of lysine) using the QuickChange^®^ Site-Directed Mutagenesis Kit from Stratagene (La Jolla, CA, USA).

The four individual K-R mutants were made separately, and are further denoted as D_4.2_R K229R, K304R, K311R, and K381R. The K304R mutant was used as a template for the construction of the quadruple mutant D_4.2_R K229,304,311,381R (further denoted as HA D_4.2 4KR_R).

HA β_2_AR was a kind gift from Prof. Dr. R. Lefkowitz (Duke University, Durham, NC). HA CXCR4 was purchased from pCDNA.org.

Primary antibodies used were mouse monoclonal anti-HA (clone16B12; Covance Research Products, cat. no. MMS-101-P-1000), rabbit polyclonal anti-Etag (Abcam, cat. no. ab3397-250), rabbit polyclonal anti-c-myc (Sigma, cat. no. C3956), rabbit polyclonal anti-HA (GeneTex, cat. no. GTX29110), rat monoclonal anti-HA (clone 3F10; Roche, cat. no. 11867423001), horseradish peroxidase (HRP)-conjugated mouse monoclonal anti-Flag M2 (Sigma, cat. no. A8592), rabbit polyclonal phospho-p44/42 MAPK (Cell Signaling, cat. no. 9101L) and mouse monoclonal p44/42 MAPK (clone L34F12; Cell Signaling, cat. no. 4696S).

Secondary antibodies used were goat anti-rabbit IRDye680 LT (cat. no. 926–68021), goat anti-mouse IRDye680 LT (cat no. 926–68020), goat anti-mouse IRDye680 RD (cat. no. 926–68070), goat anti-rabbit IRDye800 (cat. no. 926–32211), goat anti-mouse IRDye800 (cat. no. 926–32210), goat anti-rat IRDye680 LT (cat. no. 926–68029) and were all purchased from LI-COR Biosciences.

### Cell culture and transfection

HEK293T cells were cultured in Dulbecco's modified Eagle's medium (DMEM; Invitrogen), supplemented with 10% fetal calf serum, penicillin (100 U/ml), and streptomycin (100 μg/ml) in a controlled environment (37°C, 98% humidity, 5% CO2). HEK293T cells were transfected using the Polyethylenimine (PEI) method as described before [8]. A total amount of 10 μg of DNA was used for transfection of cells in one 10 cm dish.

HEK293S cells parental were a kind gift of Prof. Dr. Nico Callewaert [33]. Cells were stably transfected with calcium phosphate method with pHA D_4.2_R or pHA D_4.2 4KR_R and were grown in DMEM/F12 (Gibco, Invitrogen) supplemented with 10% fetal calf serum, penicillin (100 U/ml), streptomycin (100 μg/ml), and 0.5 mg/ml G418 (Geneticin, Gibco) in a controlled environment (37°C, 98% humidity, 5% CO2).

### Co-immunoprecipitation

Forty eight hours after transfection cells were washed twice with cold phosphate-buffered saline (PBS), harvested and the cell pellet was frozen at −70°C for at least 1 h before lysis. Cell lysates were subjected to immunoblot analysis, or to immunoprecipitation (IP) followed by immunoblotting (IB), as described before [34].

### Ubiquitination assay—sequential double immunoprecipitation

Ubiquitination assay was described before [35]. Briefly, cells were lysed in radioimmunoprecipitation buffer (RIPA) supplemented additionally with the inhibitor of deubiquitinating enzymes (10 mM), N-ethylmaleimide (NEM). For the first IP 2 μg of anti-HA (16B12) antibody was used and samples were incubated overnight with antibody and agarose beads. Next, three wash steps with the lysis buffer were performed and proteins bound to the beads (receptor and interacting partners) were eluted under denaturing conditions, and a quarter of the eluate was used to confirm the first IP. The rest of the eluates, containing denatured proteins, were diluted with lysis buffer and subjected to a second IP to remove receptor-interacting proteins from the first IP and specifically isolate the protein of interest. Finally, the eluates from the second IP were subjected to immunoblotting for the detection of ubiquitinated receptor.

### Chemical cleavage of ubiquitin with sodium hydroxide and dithiothreitol

A modified protocol of sequential double immunoprecipitation was used to detect the influence of highly reducing conditions (dithiothreitol-DTT) or high pH (NaOH) on the ubiquitination status of the receptor. High pH treatment was performed by resuspending immunoprecipitates in 0.5% SDS, boiling for 3 min, cooling to room temperature, adding NaOH to a final concentration of 50 mM, and then incubating the samples at 32°C for 1 h. Mock-treated samples were incubated with PBS. Samples were then neutralized via the addition of 0.5 M Tris/HCl, pH 6.8. 5x Laemmli sample buffer (5% SDS; 50% glycerol; 0.2% bromophenol blue; 65 mM Tris/HCl, pH 6.8) without reducing agents was added, and samples were boiled for 3 min. Next, eluates were transferred to the new tubes, diluted with lysis buffer and a second round of IP was performed as described before.

Reducing treatments were performed by resuspending immunoprecipitates in 5x Laemmli sample buffer supplemented with 100 mM DTT followed by boiling for 3 min. Next, eluates were transferred to the new tubes, diluted with the lysis buffer and a second round of IP was performed.

All protein samples were separated via SDS-PAGE, transferred to the nitrocellulose membrane and subjected to immunodetection with primary and secondary antibodies.

### Detection of p44/42 mitogen-activated protein kinase (MAPK) phosphorylation by in cell western

HEK293S cells stably transfected with HA D_4.2_R or HA D_4.2 4KR_R were grown on poly-D-lysine coated 96-well plates until 80% confluency. After overnight incubation in serum free medium, cells were treated with dopamine in concentration ranging from 10^−8.5^ to 10^−4.5^ M for 3 min at 37°C. Incubation was stopped by removing the culture medium, followed by addition of fixing solution (3.7% formaldehyde in PBS) for 20 min at room temperature (RT). Next, cells were permeabilized by washing 4 times for 5 min with Triton washing solution (0.1% Triton X-100 in PBS). Subsequently, cells were blocked with a blocking buffer (LI-COR Biosciences) for 90 min and finally, cells were incubated overnight at 4°C with two primary antibodies: rabbit phospho-p44/42 MAPK and mouse p44/42 MAPK diluted in the blocking buffer. The next day, the plate was washed 4 times for 5 min at RT with Tween washing solution (0.1% Tween-20 in PBS) and incubated with fluorescently labeled secondary antibodies: goat anti-rabbit IRDye800 and goat anti-mouse IRDye680 RD for 1 hour at RT. After final washing with Tween washing solution, fluorescent signal was detected with the Odyssey Infrared Imaging system. In the analysis, background values of the secondary antibodies are subtracted and the phospho-p44/42 MAPK signal is normalized against the total p44/42 MAPK signal.

### Immunofluorescence microscopy

HEK293S cells stably expressing HA D_4.2_R or HA D_4.2 4KR_R were seeded in wells with coverslips. Twenty four hours after seeding, cells were fixed with fixing solution (3.7% formaldehyde in PBS) for 20 min at RT. After washing, cells were permeabilized with 0.1% Triton X-100 in blocking buffer (3% newborn calf serum, 1% bovine serum albumin in PBS) for 20 min at RT. Subsequently, cells were incubated for one hour with blocking buffer, followed by incubation for one hour with rabbit anti-HA diluted in blocking buffer (1:1000). Next, secondary antibody anti-rabbit Alexa Fluor 488 diluted in blocking buffer (1:500) was applied and incubation was performed in the dark for 1 hour. Cell nuclei were stained with DAPI (1μg/ml). After each step, mentioned above, coverslips were washed three times with PBS. Mounting was performed with Mowiol and samples were analysed using the Axiocam 200 microscope (Zeiss, Thornwood, NY).

### Statistical analyses

To determine significance, results were compared with control conditions by means of *t* test (for two groups) or by One-way analysis of variance (ANOVA) with Bonferroni post-test (for more than two groups). All statistical analyses were performed using Prism software (version 5; GraphPad Software). P < 0.05 at the 95% confidence level was considered significant.

## Results

### The dopamine D_4_ receptor is ubiquitinated on non-lysine residues

In previous studies we have shown that KLHL12, a BTB-Kelch protein, functions as an adaptor in the Cullin3-based E3 ubiquitin ligase complex and promotes ubiquitination of the D_4_R [8]. In this study, we aim to characterize the ubiquitination pattern of the D_4_R into more detail.

**Fig 1:**
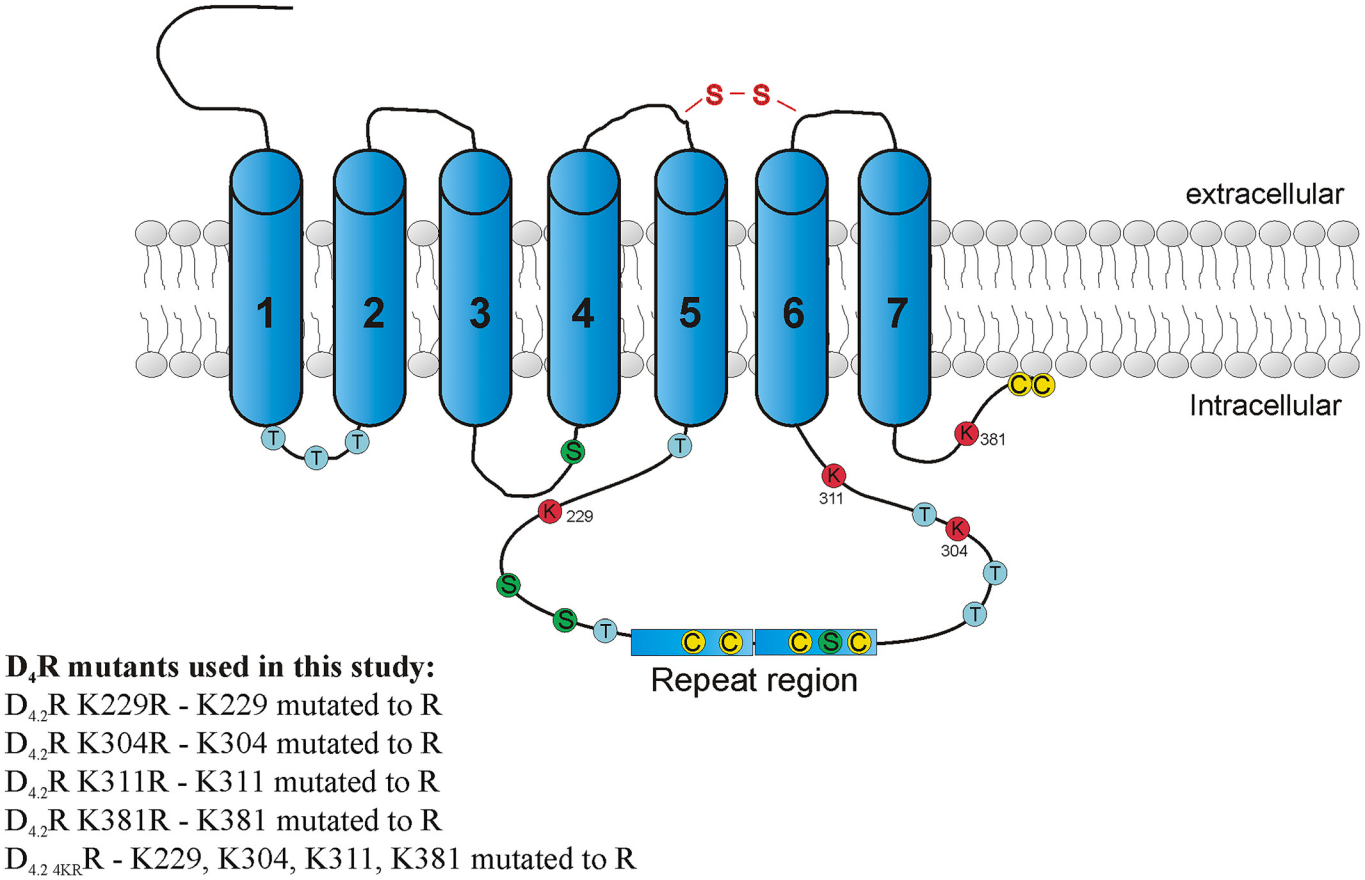
Approximate localization of all possible ubiquitin binding sites in the intracellular part of D_4.2_R. The figure shows all possible ubiquitin binding sites: lysines (K), cysteines (C), serines (S) and threonines (T) that are present in the intracellular part of the D_4.2_R. Additionally, information about receptor mutants used in this study is given.

The D_4.2_R contains only four lysine (K) residues in its whole primary structure, namely at sites 229 (in the IC3 before the polymorphic region), 304, 311 (both in the IC3 after the polymorphism) and 381 (in the C-terminal tail) that can potentially be ubiquitinated (Fig 1). In order to elucidate which lysine residue(s) could serve as acceptor site(s) for ubiquitin-conjugation, four receptor mutants, each with one of the four lysine residues mutated to arginine (R), were constructed using site-directed mutagenesis. Although the structure of an arginine residue resembles the structure of lysine, the arginine cannot serve as an acceptor site for ubiquitin-conjugation. Therefore, receptor ubiquitination is thought to be impaired upon mutation of the preferred ubiquitin-conjugation site. In parallel, we constructed a receptor mutant in which all four lysine residues were mutated (D_4.2 4KR_R) and this mutant thus should be completely unable to undergo ubiquitination. First, the expression profile of these mutants and their ability to interact with KLHL12 was tested. Upon transient transfection of the HA-tagged receptor constructs and Etag KLHL12, lysates were tested for expression of all receptors and further subjected to immunoprecipitation with anti-HA. From these experiments, it can be concluded that all K-R receptor mutants are clearly expressed, and are able to interact with KLHL12 (S1 Fig).

**Fig 2:**
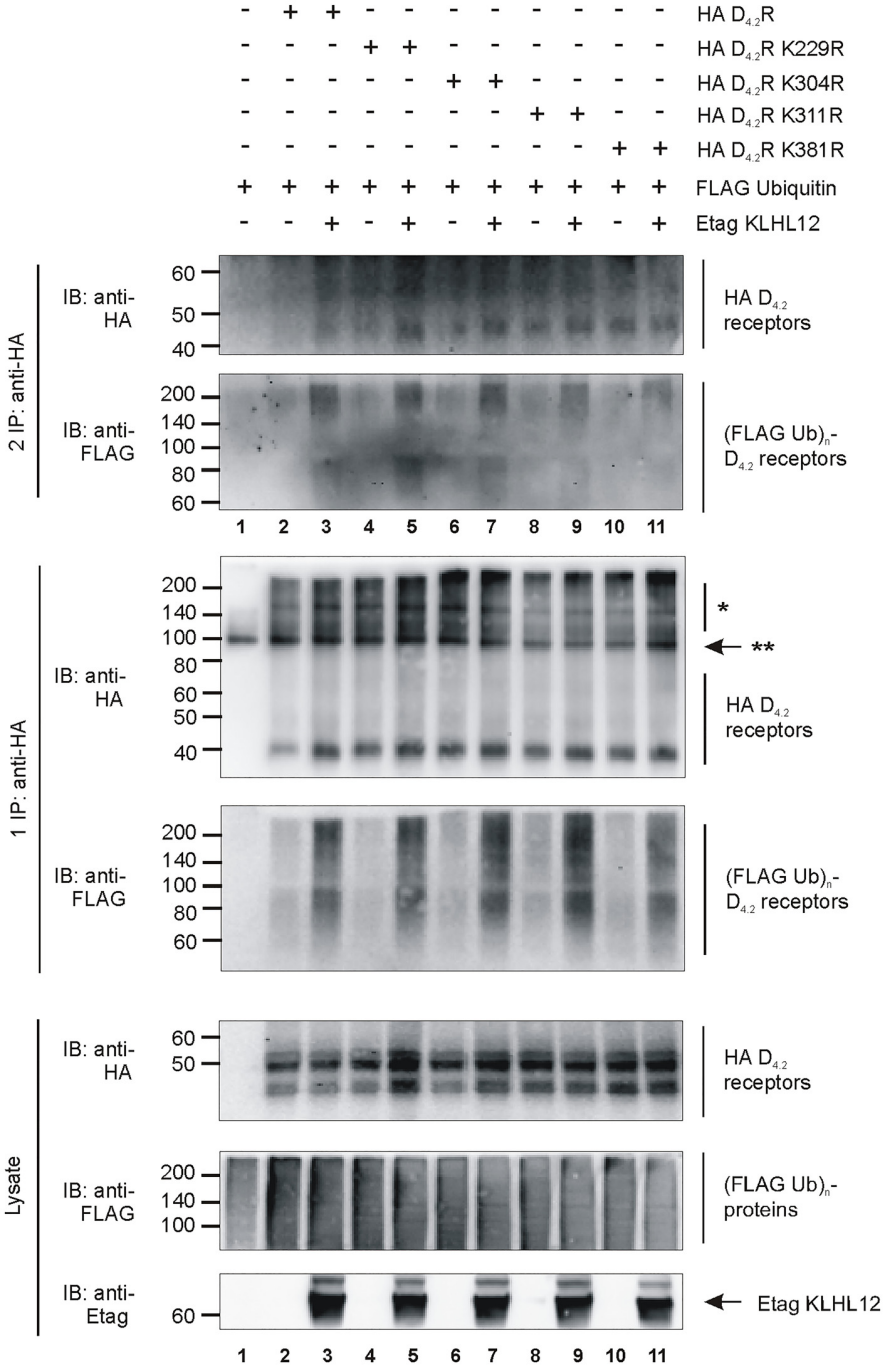
Ubiquitination of all four single K-R mutants of D_4_R is increased by KLHL12. HEK293T cells were transiently transfected as indicated. 48 h post-transfection, cells were harvested and lysed. 5% of the lysate was used for IB to visualize HA-tagged WT D_4.2_R and all single K-R mutants, (Flag Ub)n-proteins, and Etag KLHL12, respectively (bottom panels). The rest of the lysate was subjected to a sequential double IP with anti-HA (16B12). Specific purification of the HA D_4.2_R WT and all K-R mutants was confirmed after the first (middle panels) and second IP (upper panels) upon IB with anti-HA (16B12, 1:2000), whereas receptor ubiquitination was revealed upon IB with anti-Flag-HRP (1:2000). * D_4_R specific signal. ** Association of two heavy chains of mouse anti-HA antibody (each 50 kDa). The results shown are representative of three independent experiments.

Subsequently, ubiquitination of all individual mutant receptors was tested by ubiquitination assays in HEK293T cells (Fig 2). Surprisingly, comparable ubiquitination levels could be observed for all four single K-R mutant receptors (lanes 4, 6, 8, 10). KLHL12 promoted ubiquitination of the WT HA D_4.2_R (lane 3) but also of all four single K-R mutant receptors (1 and 2 IP: lanes 5, 7, 9, 11). These observations could reflect the possibility that upon mutation of one lysine residue, another lysine residue serves as an alternative acceptor site for ubiquitin-conjugation. Therefore, these results suggest redundancy of the lysine residues in the D_4_R to serve as attachment sites for basal or KLHL12-promoted ubiquitination.

**Fig 3:**
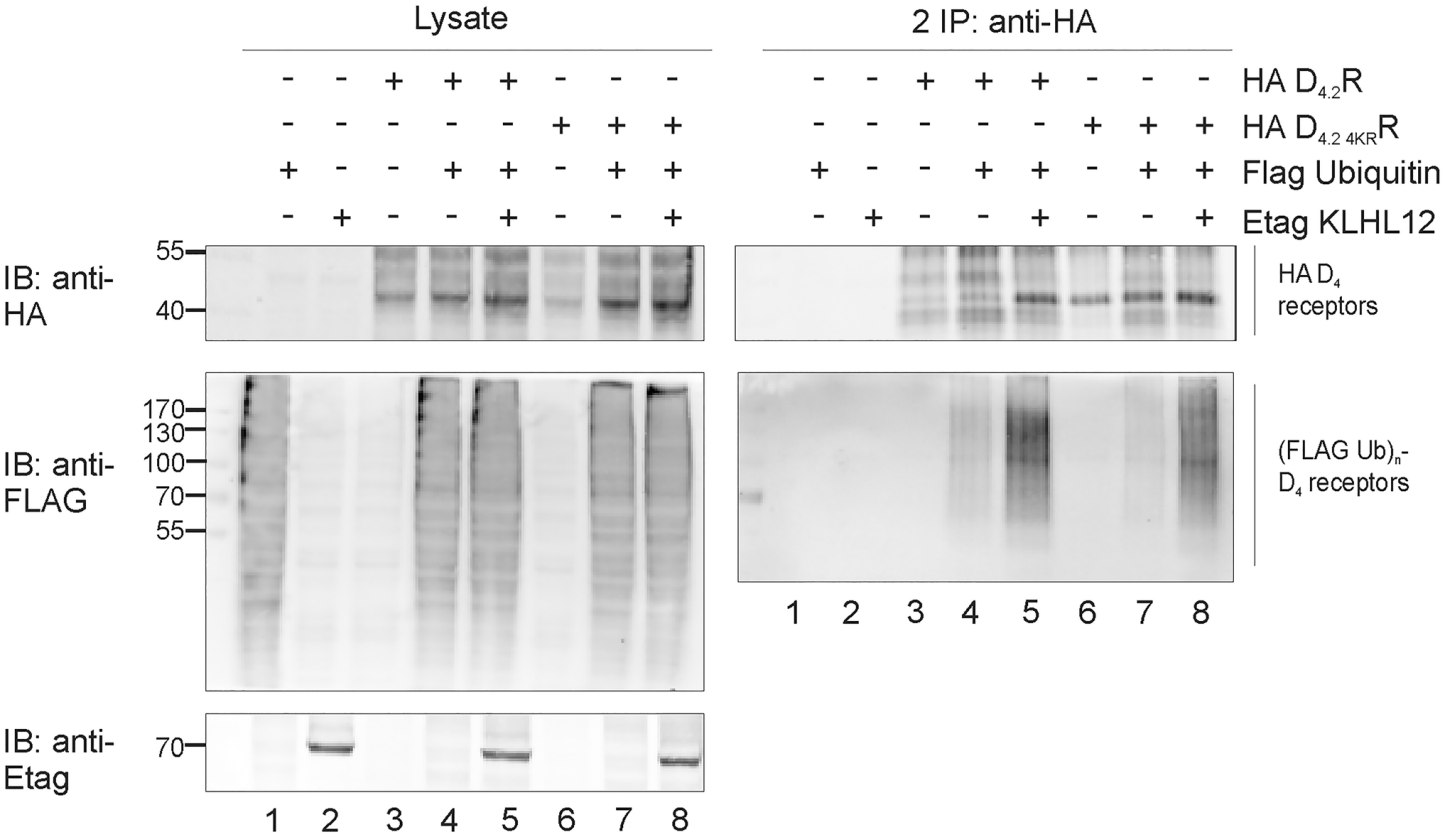
Lysine lacking D_4.2 4KR_R mutant is ubiquitinated and KLHL12 enhances its ubiquitination. HEK293T cells were transiently transfected as indicated. 48 h post-transfection, cells were harvested and lysed. 5% of the lysate was used for IB to visualize HA D_4_R, (Flag Ub)n-proteins, and Etag KLHL12, respectively (left panels). The rest of the lysates were subjected to a sequential double IP with anti-HA (16B12). Specific purification of the receptor after the second IP was confirmed upon IB with rat anti-HA (1:2000), whereas receptor ubiquitination was revealed upon IB with anti-Flag-HRP (right panels, 1:2000). The results shown are representative of four independent experiments.

In the next step, the membrane expression and functionality of the quadruple HA D_4.2 4KR_R mutant was verified by examining its ability to phosphorylate MAP kinase in response to agonist stimulation (S2 Fig). In a second stage, the ubiquitination level of this mutant receptor was investigated. In these experiments, a very strong increase in ubiquitination of the mutant receptor was again detected in the ubiquitination assay upon overexpression of Etag KLHL12 (Fig 3) which suggests that D_4_R can be ubiquitinated on other residues than lysine.

Up to now, besides on lysine, ubiquitination on the N-terminus [11–13], on cysteine [14–16], on serine and threonine [17–19] was described. Because of its extracellular location, KLHL12-mediated ubiquitination of the N-terminus of D_4_R is unlikely. Therefore, in further investigations we focused only on the possible ubiquitination of the residues which are located in the intracellular parts of the receptor. Fig 1 shows the estimated localization of all possible ubiquitination sites in the intracellular part of the D_4_R.

### KLHL12 can promote ubiquitination on cysteine residues of D_4_R

**Fig 4:**
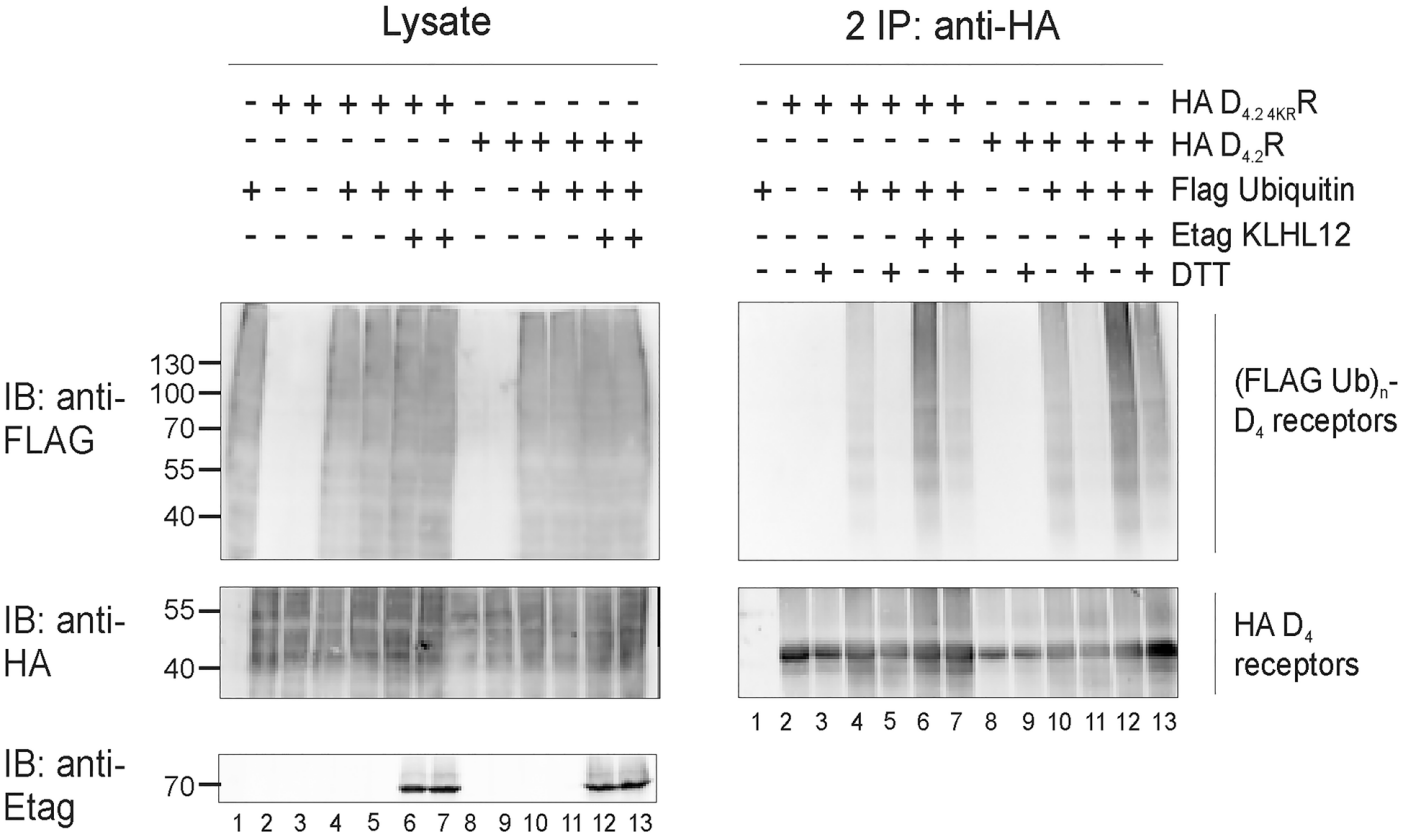
Treatment with a highly reducing agent decreases ubiquitination of D_4.2_R WT and D_4.2 4KR_R mutant. HEK293T cells were transiently transfected as indicated. 48 h post-transfection, cells were harvested and lysed. 5% of the lysates were used for IB to visualize (Flag Ub)n-proteins, HA D_4_R, and Etag KLHL12, respectively (left panels). The rest of the lysates were subjected to IP with anti-HA (16B12). After IP proteins were eluted at 95°C with or without addition of 100 mM DTT. Next, a second round of IP with anti-HA antibody was performed. Specific purification of the receptor after the second IP was confirmed upon IB with rat anti-HA (1:2000), whereas receptor ubiquitination was revealed upon IB with anti-Flag-HRP (right panels, 1:2000). Shown are results from a single experiment, representative of three independent experiments.

As shown in Fig 1 there are two conserved cysteine residues in each polymorphic repeat in the third intracellular loop and an additional two are present at the C-terminus of D_4_R. To investigate a possible attachment of ubiquitin to cysteine we decided to use a specific chemical approach, in which we rely on the fact that the thioester bond which is formed between ubiquitin and cysteine is susceptible to strong reducing conditions (for example high concentration of DTT). For this assay, HA-tagged WT D_4.2_R or mutant D_4.2 4KR_R were immunoprecipitated from transiently transfected HEK293T cells. The resulting IP pellets containing ubiquitinated receptors were treated with 100 mM DTT, which has been shown to cleave Ub chains attached to cysteine residues [15, 16, 36]. Next, eluates were diluted with lysis buffer and a second IP was performed, as before, to prevent detection of a ubiquitination signal originating from D_4_R interacting proteins instead of the receptor itself (Fig 4). Treatment with the strong reducing agent DTT after performing the ubiquitination assay resulted in a very strong decrease in ubiquitination signal of WT D_4.2_R and of the lysine-deficient D_4.2 4KR_R mutant. When Etag KLHL12 was overexpressed, the detected ubiquitination signal was much stronger also in the samples treated with DTT (e.g. compare in Fig 4 lane 11 and lane 13 for WT D_4.2_R and compare lane 5 and 7 for the mutant D_4.2 4KR_R), but DTT treatment again decreased the ubiquitination signal very efficiently. These results indicate that KLHL12 can promote cysteine-linked D_4_R ubiquitination. The ubiquitination signal is still visible after DTT treatment in case of D_4.2 4KR_R when Etag KLHL12 is overexpressed and therefore, these results suggest that there are also other residues, besides cysteine and lysine, that can be ubiquitinated in the D_4_R

### KLHL12 promotes ubiquitination on serine and/or threonine residues of D_4_R

**Fig 5:**
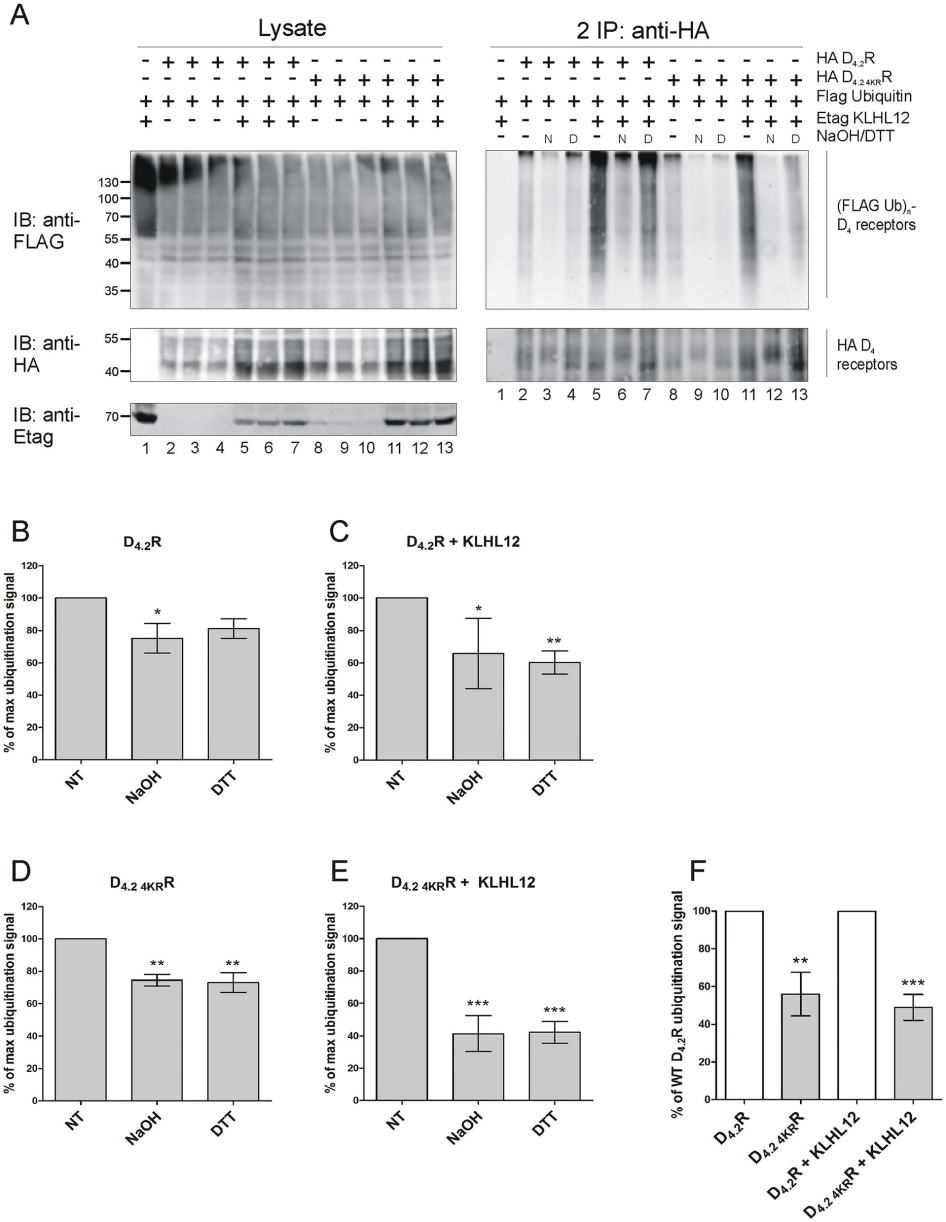
Treatment with NaOH decreases ubiquitination of D_4.2_R WT and D_4.2 4KR_R mutant. A) HEK293T cells were transiently transfected as indicated. 48 h post-transfection, cells were harvested and lysed. 5% of the lysates were used for IB to visualize (Flag Ub)n-proteins, HA D_4_R, and Etag KLHL12, respectively (left panels). The rest of the lysates were subjected to IP with anti-HA (16B12). After IP proteins were eluted in 0.5% SDS at 95°C. Next, lysates were incubated with or without addition of 50 mM NaOH for 1 h at 32°C, mock treated samples were incubated with PBS. Additionally, samples were treated with a highly reducing agent (5 min, 95°C, 100 mM DTT). After dilution with RIPA lysis buffer a second round of IP was performed with anti-HA antibody. Specific purification of the receptor after the second IP was confirmed upon IB with rat anti-HA (1:2000), whereas receptor ubiquitination was revealed upon IB with anti-Flag-HRP (right panels, 1:2000). N: samples treated with 50 mM NaOH, D: samples treated with 100 mM DTT. Experiment with two different chemical treatments was performed three times. Additionally, two more experiments were performed with NaOH treatment alone. B-E) Quantification of the ubiquitination signal detected in four to seven independent experiments was performed using Fiji software. Values were normalized to the receptor level in the corresponding lanes. Graph bars show percentage of maximal ubiquitination signal (mean ± SEM) where maximum is equal to 100% and represents the signal detected in samples which did not undergo any chemical treatment (NT- non-treated) in each independent experiment. Compared with NT: ***, P < 0.001; **, P < 0.01; *, P < 0.05. F) Quantification of the ubiquitination signal detected in five to seven independent experiments was performed using Fiji software. Values were normalized to the receptor level in the corresponding lanes. Graph bars show percentage of ubiquitination signal detected for WT D_4.2_R (mean ± SEM). Compared with the ubiquitination signal detected for WT D_4.2_R without or with overexpression of KLHL12, respectively: ***, P < 0.001; **, P < 0.01.

When ubiquitin is attached to serine or threonine an oxyester bond is formed. This kind of bond can be destroyed by treatment with a strong base (50–100 mM NaOH) [36, 37]. To examine the possibility of ubiquitination of the D_4_R on serine and threonine residues, IP eluates were treated with 50 mM NaOH for 1h at 32°C as described before [37] (Fig 5A). In the same experiment we also examined the influence of highly reducing conditions on the same samples to remove ubiquitin from cysteine residues (as described above). This facilitated the comparison of the results of the two different chemical treatments, indicative for serine/threonine and cysteine ubiquitination, respectively. After the first IP samples were diluted with lysis buffer and a second IP was performed similar as before.

As shown in Fig 5A treatment with NaOH led to a significant decrease in basal ubiquitination as well as a strong decrease in KLHL12-enhanced ubiquitination signal in case of both WT D_4.2_R and D_4.2 4KR_R mutant. In this experiment the influence of DTT can also be clearly observed. Independent ubiquitination assays upon treatment with NaOH and DTT were performed at least four times for each of the treatments and we consistently observed a clear decrease in ubiquitination signal. However, often the receptor level after the second IP was not equal in all of the samples and therefore, we decided to quantify the detected ubiquitination signal and normalize it against the receptor levels to identify trends in obtained data (Fig 5B–5E). The results presented in Fig 5A suggest that NaOH is more potent than DTT in removing ubiquitin moieties from D_4_R, although the performed quantification from two to seven independent experiments does not support this observation. However, and most importantly, all these results together suggest that ubiquitination of cysteine, serine and/or threonine residues in the D_4_R is possible. Moreover, from this quantification we can conclude that the decrease in ubiquitination signal caused by the chemical treatments is bigger in case of KLHL12-enhanced ubiquitination especially for the D_4.2 4KR_R (compare bars representing percentage of maximal ubiquitination signal detected upon treatment in Fig 5B and 5C for WT D_4.2_R and Fig 5D and 5E for D_4.2 4KR_R). Another interesting observation that can be made based on experiments involving chemical treatments is the overall lower ubiquitination level of D_4.2 4KR_R mutant compared to the WT D_4.2_R (Fig 5F). This suggests that lysines which are present in WT receptor can also be ubiquitinated and represent a fraction of the total ubiquitination signal, but it is not possible to conclude whether they are the preferred ubiquitin-binding sites in the KLHL12-promoted ubiquitination. As suggested by Wang et al. [38] before making a conclusion about non-lysine ubiquitination based on chemical treatment experiments one should be aware that too long alkaline treatment can affect also amide bonds formed between lysine and ubiquitin. Therefore, an additional control experiment was performed in which the influence of alkaline treatment on known lysine-linked ubiquitination samples was tested. We have examined the effect of NaOH treatment on ubiquitination of two other GPCRs for which ubiquitination on lysine residues was described, namely the chemokine CXCR4 receptor [26, 39] and the β_2_-adrenoceptor (β_2_AR) [28]. S3 Fig clearly demonstrates that NaOH strongly affects basal and KLHL12-enhanced ubiquitination of D_4_R but seems to have no effect on ubiquitination of the other two receptors. This indicates that the NaOH treatment conditions used in our experiments are not strong enough to affect typical isopeptide bonds which are formed between ubiquitin and lysine residues in substrate proteins.

### KLHL12 promotes differential ubiquitination of the D_4_R polymorphic variants

As mentioned in the introduction D_4_R has an important polymorphism in the third intracellular loop where a 16-amino acid sequence is repeated from 2 to 11 times. The most common variants in the human population are D_4.4_R (64%), D_4.7_R (21%) and D_4.2_R (8%), respectively [4]. Not much is known about functional differences between different polymorphic variants but several studies suggest an association between the D_4.7_R allele and ADHD [6].

Previously, we have demonstrated that KLHL12 binds specifically to the polymorphic region of D_4_R and promotes ubiquitination of the receptor. For these reasons, we were interested to explore whether KLHL12 differentially binds to the most frequent polymorphic variants and to study the effect of KLHL12 on the ubiquitination of these receptor variants.

**Fig 6:**
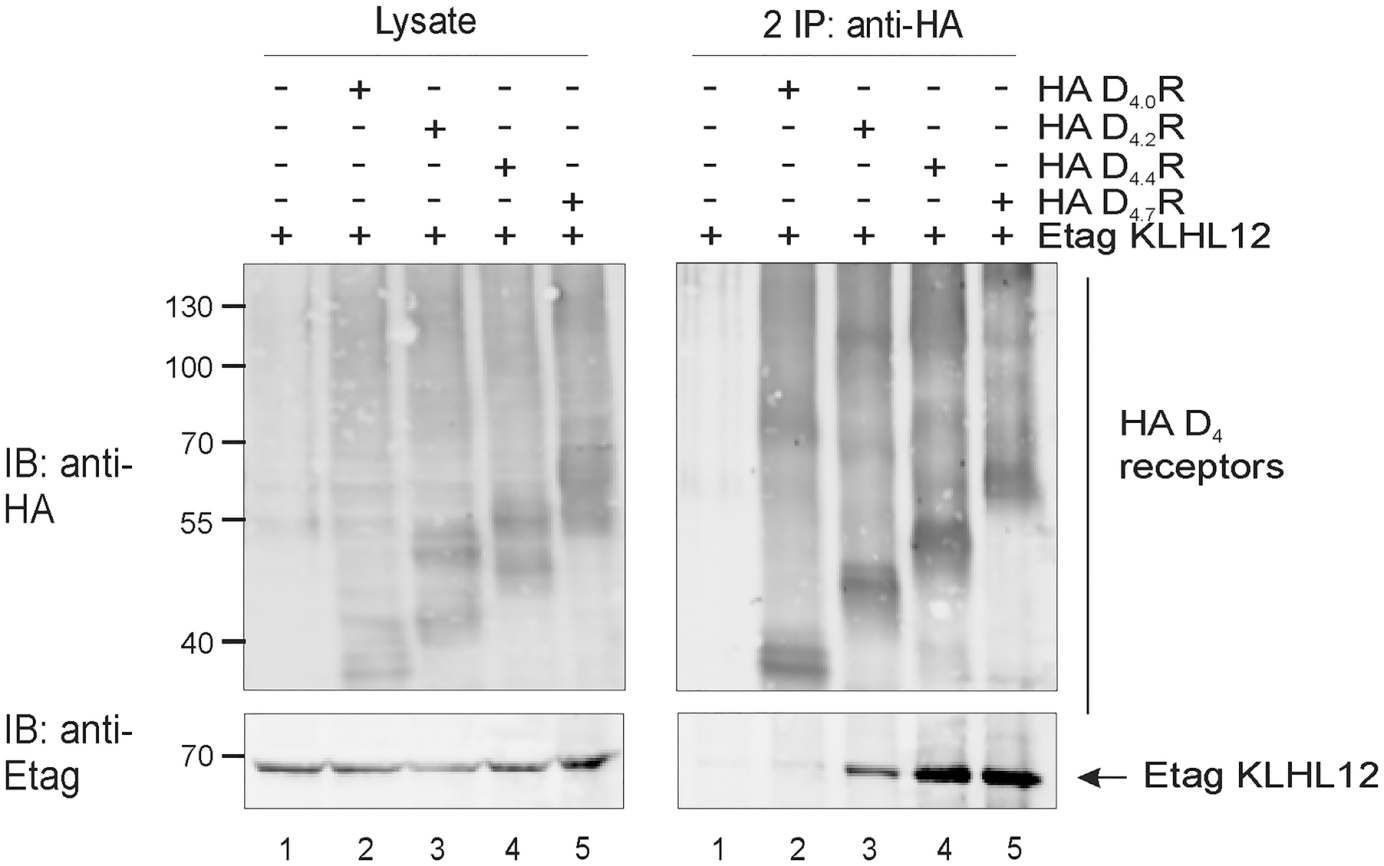
All common D_4_R polymorphic variants interact with KLHL12 while D_4.0_R, lacking repeats in the third intracellular loop, does not interact. HEK293T cells were transiently transfected as indicated. 48 h post-transfection, cells were harvested and lysed. Part of the lysate was used for IB to verify expression of HA-tagged D_4_R variants and Etag KLHL12 (left panels). The rest of the lysate was subjected to IP with anti-HA (16B12). Specific purification of the receptor after IP was confirmed upon IB with rat anti-HA (1:2000). Interaction of Etag KLHL12 with the D_4_R polymorphic variants was verified by IB with anti-Etag (1:2000). Data shown are representative of three independent experiments. Expected molecular weights of different D_4_R variants: D_4.0_R (44–50 kDa), D_4.2_R (47-53kDa), D_4.4_R (51–57 kDa), D_4.7_R (56–62 kDa).

First, we have examined using co-immunoprecipitation if KLHL12 binds to all common D_4_R polymorphic variants, namely D_4.2_R, D_4.4_R and D_4.7_R. As a negative control we used an artificial receptor variant from which the polymorphic region was removed (D_4.0_R). Fig 6 clearly shows that KLHL12 binds to all common polymorphic variants D_4.2_R, D_4.4_R and D_4.7_R and as expected no interaction with D_4.0_R was detected.

**Fig 7:**
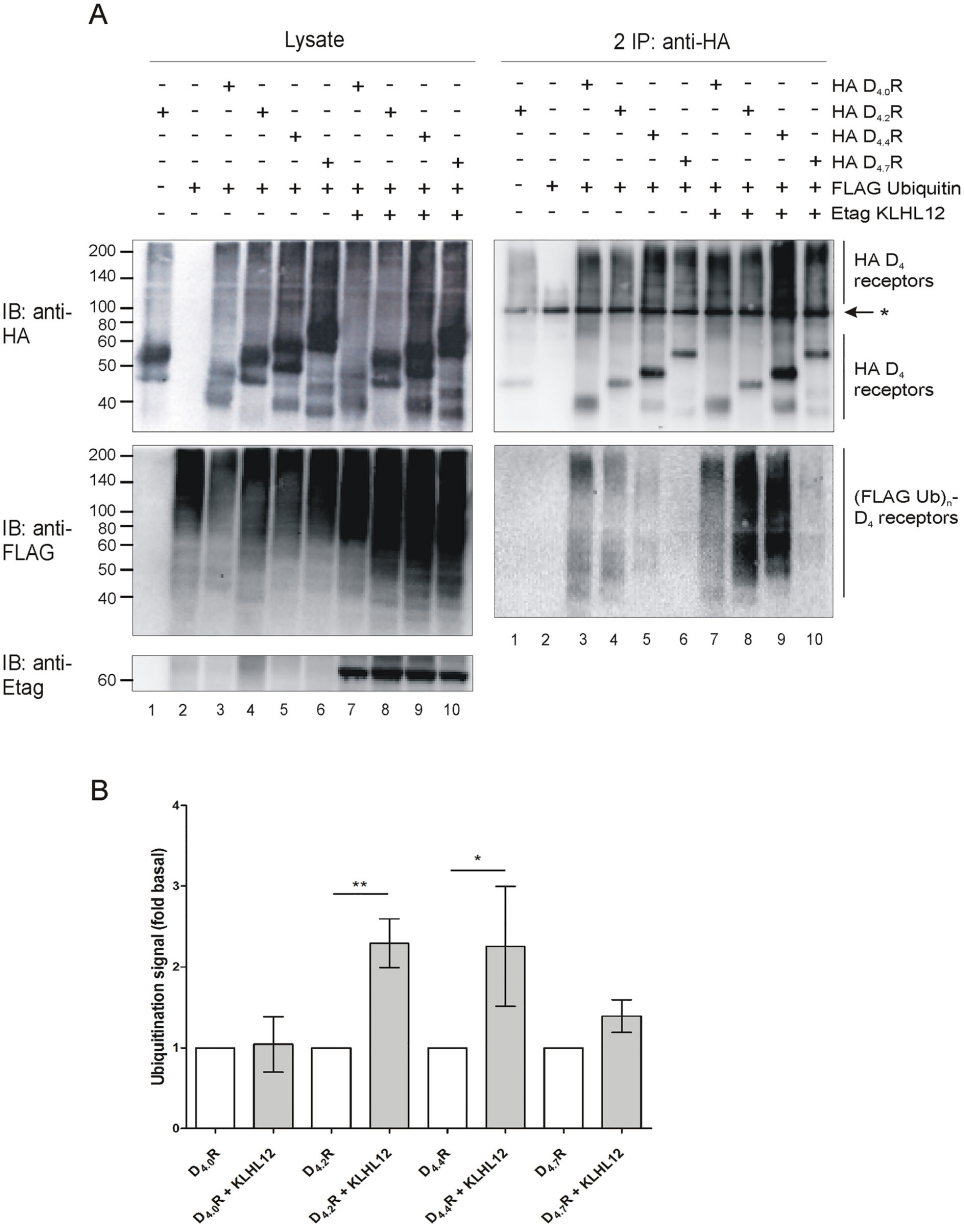
KLHL12 strongly enhances ubiquitination of D_4.2_R and D_4.4_R but hardly of D_4.7_R. A) HEK293T cells transiently transfected as indicated were lysed 48 h post-transfection. 5% of the lysate was used for IB to verify expression of HA-tagged D_4_R polymorphic variants, (Flag Ub)n-proteins and Etag KLHL12 (left panels). The rest of the lysate was used for double sequential IP with anti-HA (16B12). Specific purification of the receptors after the second IP was confirmed upon IB with mouse anti-HA (16B12, 1:2000), whereas receptor ubiquitination was revealed upon IB with anti-Flag-HRP (right panels, 1:2000). * Association of two heavy chains of mouse anti-HA antibody (each 50 kDa). Data shown are representative of similar results from five independent experiments. B) Quantification of the ubiquitination signal detected in three to five independent experiments was performed using Fiji software. Values were normalized to the receptor level in the corresponding lanes. Graph bars show ubiquitination signal (mean ± SEM, fold basal) where basal ubiquitination represents the signal detected in samples without overexpression of KLHL12, for the specific D_4_R variant, in each independent experiment. Compared with the D_4.X_ R without KLHL12 overexpression: **, P < 0.01; *, P < 0.05.

Next, ubiquitination assays were performed in HEK293T cells overexpressing different HA-tagged D_4_R polymorphic variants with or without co-expression of Etag KLHL12. A strong increase in ubiquitination signal was observed in case of D_4.2_R and D_4.4_R variants when Etag KLHL12 was overexpressed. Surprisingly, we could hardly detect an increase in ubiquitination of the D_4.7_R variant (Fig 7).

**Fig 8:**
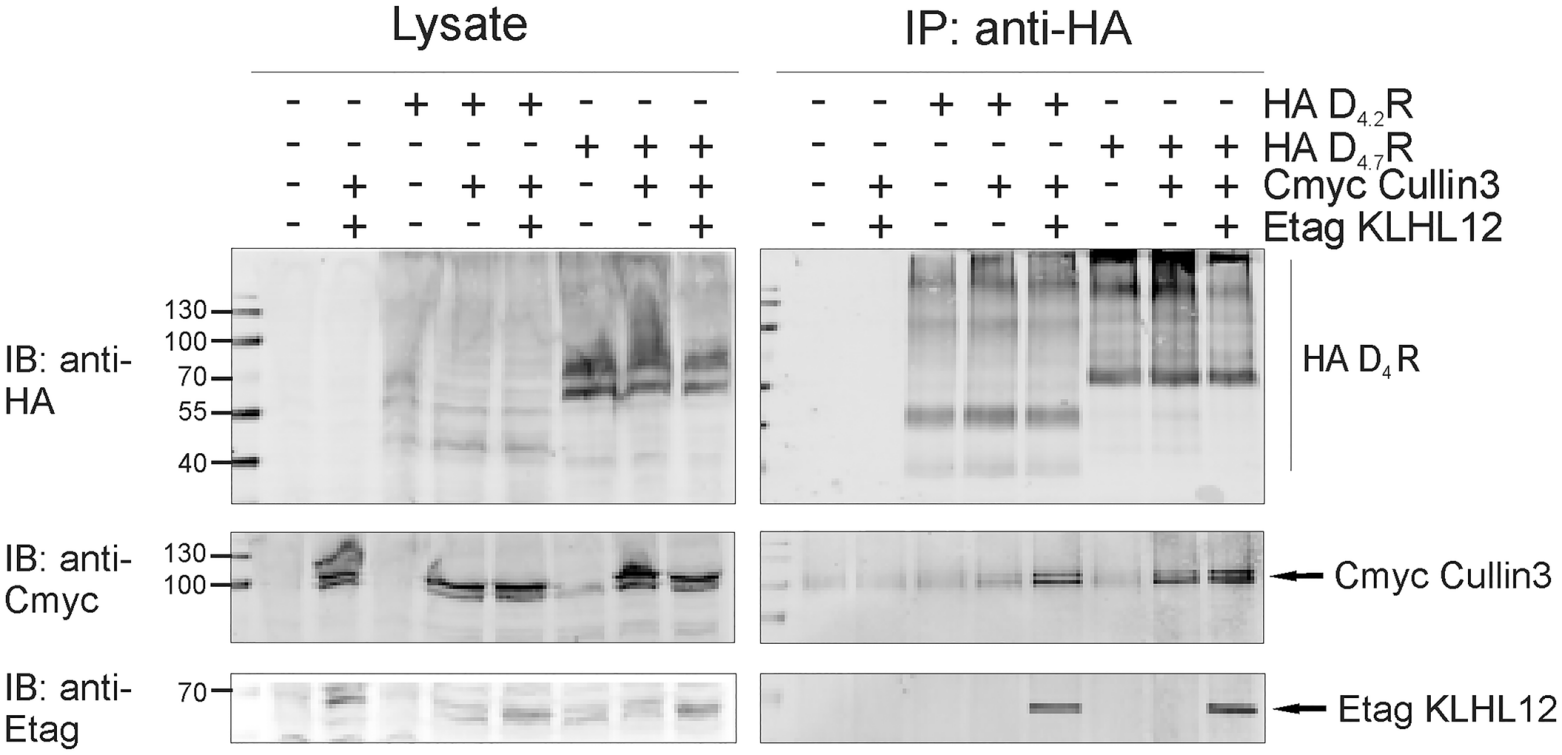
D_4.7_R forms a complex with Cullin3. HEK293T cells were transiently transfected as indicated. 48 h post-transfection, cells were harvested and lysed. 5% of the lysate was used for IB to verify expression of HA-tagged receptors, Cmyc Cullin3 and Etag KLHL12 (left panels). The rest of the lysate was subjected to IP with anti-HA (16B12). Specific purification of the receptor after IP was confirmed upon IB with rat anti-HA (1:2000). Interaction of Etag KLHL12 with D_4_R was verified by IB with anti-Etag (1:2000) and the presence of Cmyc Cullin3 was visualized with anti-Cmyc (right panels, 1:1000).

The ubiquitination process requires integrated action of many proteins at the same time and therefore, we have also examined if the D_4.7_R variant is still capable to form a complex with E3 ligase, Cullin3. The results from the co-immunoprecipitation studies indicate that D_4.7_R is able to form a complex with Cullin3 (Fig 8), although we cannot rule out the possibility that this interaction could be mediated by adaptor proteins other than KLHL12.

## Discussion

Previously, we have shown that KLHL12 interacts with the polymorphic repeats of the D_4_R and enhances its ubiquitination [8] but this has no influence on receptor degradation [31]. In the present study we investigated which specific residues of the D_4_R are ubiquitinated by the KLHL12-Cullin3 E3 ligase complex. In the whole sequence of D_4_R there are only four lysine residues and all of them can potentially serve as ubiquitin binding sites as they are localized in the intracellular domains of the receptor. Using a site-directed mutagenesis approach each lysine was separately mutated to arginine. Next, sequential double IP experiments demonstrated that all four mutants are ubiquitinated and overexpression of KLHL12 increases their ubiquitination level (Fig 2). This suggests that ubiquitination occurs on multiple lysines or that the ubiquitination system is flexible and when the most preferred ubiquitin binding site is unavailable it can ubiquitinate another available lysine. Next, we created the quadruple mutant HA D_4.2 4KR_R in which all four lysines were mutated to arginines. This mutant should be unable to undergo lysine-mediated ubiquitination. Surprisingly, we could still detect basal ubiquitination of this mutated receptor and upon co-expression of KLHL12 an increase in its ubiquitination was visualized (Fig 3). This finding encouraged us to search for other possible ubiquitination sites in the D_4_R. Ubiquitination on non-lysine residues is still a poorly studied phenomenon and until now ubiquitination on the N-terminus [11–13, 40], cysteine [14–16], serine and threonine [17–19] residues was described, but it has only been suggested once for a GPCR unfortunately without characterizing the precise amino acid residue [32]. In our study we investigated the possibility of ubiquitin binding to cysteine, serine and threonine residues within the intracellular domains of the D_4_R. One of the most common approaches to verify ubiquitination on a non-lysine residue is a chemical cleavage of ubiquitin [38]. Ubiquitin attached to a lysine residue forms an isopeptide bond which is very stable, whereas a thioester bond connecting ubiquitin with cysteine can be destroyed during treatment with a highly reducing agent e.g. a high concentration of DTT [15, 16, 36]. Our results clearly indicate that upon treatment with a very high concentration of DTT the ubiquitination signal detected after the second IP is significantly reduced for both WT D_4.2_R and D_4.2 4KR_R, suggesting ubiquitination on cysteine residues (Fig 4).

Next, we examined the possibility of ubiquitination on serine and/or threonine residues. When ubiquitin is attached to a serine or threonine residue an oxyester bond is formed which is susceptible for cleavage in a strong alkaline environment [36, 37]. We have performed a ubiquitination assay in which, after the first IP, the samples were treated with NaOH and after the second IP a strong decrease in ubiquitination signal was observed (Fig 5A). These results thus suggest that D_4_R ubiquitination on cysteine, serine and/or threonine residues can be promoted by KLHL12. It is, however, not possible to say which residues are the most preferred for KLHL12-induced ubiquitination as quantification of the results from multiple independent experiments indicates a comparable decrease in ubiquitination levels for both types of treatments (Fig 5B–5E). The decrease is more explicit when Etag KLHL12 is co-expressed which suggests that during KLHL12-promoted ubiquitination more non-lysine residues in the D_4_R are ubiquitinated compared to the basally ubiquitinated receptor. This decrease is even more striking in case of the D_4.2 4KR_R mutant in which no lysine residues are available. This indicates that in the WT receptor KLHL12 promotes ubiquitination on lysine and non-lysine residues and in the D_4.2 4KR_R mutant, in which lysines are not available, only non-lysine residues are ubiquitinated. The fact that in all experiments we detect an overall lower ubiquitination signal from D_4.2 4KR_R (Fig 5F) compared to WT D_4.2_R further supports the hypothesis about the involvement of lysine residues in KLHL12-mediated ubiquitination of D_4_R. To exclude the possibility that the alkaline treatment affects amide bonds which are normally formed between ubiquitin and lysine residues we have also checked the influence of NaOH on ubiquitination of other GPCRs for which lysines were described as ubiquitin binding sites. We have used the CXCR4 receptor for which three lysines in the C-terminal tail were shown to be critical for receptor ubiquitination [26]. As a second receptor we used the β_2_AR for which lysines in the third intracellular loop and in the C-terminal tail were presented to be important for ubiquitination and subsequent lysosomal degradation [28]. In this control experiment (S3 Fig), we have seen again a very strong effect of NaOH treatment on basal and KLHL12-induced ubiquitination of D_4.2_R and no effect on CXCR4 and β_2_AR ubiquitination. This result allows us to conclude that the conditions used for alkaline treatment are not affecting amide bonds which are formed during the typical lysine-linked ubiquitination process. Most papers that study ubiquitination on non-lysine residues identified several different residues as possible ubiquitination sites. Some of them are most preferred by the ubiquitination machinery but when those are not available ubiquitin can bind to other amino acids. Such situation was described for example for NS-1 nonsecreted immunoglobulin light chain (NS-1 LC) which is predominantly ubiquitinated on serine/threonine residues but when these amino acids were mutated then lysine residues could serve as ubiquitin-binding sites. Mutation of all three types of amino acids (serines, threonines and lysines) was required to efficiently reduce ubiquitination and stabilize the NS-1 LC [37]. Another example represents the T-cell antigen receptor α-chain (TCRα) for which mutation of two conserved serine residues to alanines in the cytosolic tail inhibited significantly receptor ubiquitination. However, when serine residues were replaced with lysine, cysteine or threonine residues the protein was still ubiquitinated [17]. Our findings seem to be in agreement with this previously described phenomenon showing that the ubiquitination machinery is a very flexible system and can easily change from one to another ubiquitination site if necessary.

To obtain a direct proof of ubiquitination on a non-lysine residue we decided to perform mass spectrometry (MS) analysis. Detection of ubiquitination by MS is possible due to the di-glycine remnant that remains attached to the ubiquitinated residue after tryptic digestion [41]. We have performed MS/MS analysis using a Fourier transform mass spectrometer but no ubiquitination was detected. Unfortunately, during tryptic digestion D_4_R is cut to single amino acid or very large fragments making it very hard to analyze. The computational prediction of cleavage of D_4_R with other enzymes, commonly used to prepare samples for MS analysis, did not suggest any good alternative to trypsine that could resolve this problem. According to our knowledge non-conventional ubiquitination was never proved with MS analysis probably due to the labile nature of thio-/oxy-ester bonds which are formed between ubiquitin and non-lysine residues. So far, only one study described the use of a novel peptide-based SILAC method to identify non-conventional ubiquitination of T-cell receptor α [42]. The authors could identify modified peptide which did not contain lysine in its sequence, however, they were unable to pinpoint the specific residue that undergoes ubiquitination.

Therefore, it is not very surprising that the detection of ubiquitination of D_4_R with MS/MS was not successful.

However, and most importantly, all the obtained data allow us to conclude that KLHL12 can promote ubiquitination of D_4_R on non-lysine residues and that ubiquitination on cysteine, serine and/or threonine is possible. The functional role of this non-lysine ubiquitination is hard to study because it would require the creation of a receptor with all possible ubiquitination sites mutated. Mutation of all intracellular cysteines, serines and threonines increases the chances of disrupted protein folding and it can also interfere with other posttranslational modifications. GPCRs are tightly regulated by phosphorylation which mainly occurs on serine and threonine residues and also by palmitoylation on cysteine residues (conserved cysteines located on the C-terminus of many GPCR) and also D_4_R shows these posttranslational modifications. Additionally, this can also inhibit binding of some interacting proteins, e.g. β-arrestins for which often phosphorylation of the receptor is required and which also play a critical role in the regulation of GPCR signaling. Therefore, mutation of all of these residues increases the possibility of creating of a mutant with completely different signaling properties compared to the wild type receptor. In most of the examples of non-lysine ubiquitination, which were described up to now, this modification seems to serve as a signal for protein degradation [14, 17–19, 21]. Only in the case of Pex5p receptor (cytosolic receptor for peroxisome matrix proteins) ubiquitination of conserved cysteine^11^ was shown to be involved in regulation of the recycling of the receptor form the peroxisomes to cytosol [15]. To the best of our knowledge, ubiquitination of the non-lysine residue was suggested only once for a GPCR. The group of Shenoy has shown that β_2_AR is probably ubiquitinated on a non-lysine residue in response to the treatment with an antagonist, carvedilol, as they could still detect ubiquitination of the β_2_AR in which lysines were mutated. Interestingly, the same mutant was not ubiquitinated upon agonist, isoproterenol, treatment. Carvedilol-promoted ubiquitination is mediated by a completely different E3 ubiquitin ligase which is not involved in the, already well defined, lysine-mediated ubiquitination of β_2_AR. However, both types of ubiquitination lead to endocytosis and lysosomal sorting of the receptor [32].

In the second part of our study we investigated the ubiquitination status of the three most common D_4_R polymorphic variants. First, the interaction of KLHL12 with these variants was examined. After IP of HA-tagged receptors co-precipitation of Etag KLHL12 with D_4.2_R, D_4.4_R and D_4.7_R was detected (Fig 6). As a negative control we used D_4.0_R which is an artificial receptor variant lacking the polymorphic repeats. Next, a double sequential IP was performed to investigate the ubiquitination status of the different polymorphic variants. Ubiquitination of D_4.2_R and D_4.4_R was clearly increased when Etag KLHL12 was co-expressed, but surprisingly we hardly observed increased D_4.7_R ubiquitination (Fig 7A and 7B), although this receptor variant is still able to form a complex with KLHL12 and Cullin3 (Fig 8). The differential ubiquitination pattern of the D_4.7_R, compared to the other D_4_R variants, is highly interesting as until now there is hardly evidence for pharmacological differences between this variant and the other receptor variants [43–45], although behavioral research showed that the D_4.7_R is linked with a predisposition to develop ADHD [6], and also its association with increased sexual behavior, alcohol and cigarette craving was suggested. The functional role of differential KLHL12-mediated ubiquitination of the D_4_R is still under investigation. This finding opens a lot of new questions for future research on the possible role of ubiquitination in receptor signaling and suggests that ubiquitination can regulate GPCRs in a much more complicated way than previously expected.

## Supporting Information

S1 Fig All K-R D_4_R mutants interact with KLHL12. HEK293T cells were transiently transfected as indicated. 48 h post-transfection, cells were harvested and lysed. 5% of the lysate was used for IB to visualize HA-tagged D_4_ receptors and Etag KLHL12. The rest of the lysate was subjected to IP with anti-HA (16B12). Specific purification of the receptors after IP was confirmed upon IB with anti-HA (1:2000), whereas co-precipitation of Etag KLHL12 was verified by IB with anti-Etag (1:2000). * Association of two heavy chains of mouse anti-HA antibody (each 50 kDa).
(TIF)

S2 Fig D_4.2 4KR_R is expressed on the membrane and its stimulation leads to the phosphorylation of p44/42 MAPK. A) HEK293S cells stably expressing HA D_4.2_R and HA D_4.2 4KR_R were treated with dopamine in concentration ranging from 10^−8.5^ to 10^−4.5^ M. An *in cell western* assay was performed and phosphorylated p44/42 MAP kinase was detected with rabbit anti-phospho p44/42 MAPK (1:1000). Values were normalized against the signal detected with mouse anti-p44/42 MAPK (1:800). B) HEK293S cells stably expressing HA D_4.2_R and HA D_4.2 4KR_R were seeded in wells with coverslips. Cells were stained using primary antibody rabbit anti-HA (1:1000) and secondary antibody anti-rabbit Alexa Fluor 488 (1:500). Cell nuclei were colored with DAPI.
(TIF)

S3 Fig Treatment with NaOH decreases ubiquitination of D_4.2_R but has no influence on ubiquitination of CXCR4 receptor or β_2_-adrenoceptor (β_2_AR). HEK293T cells were transiently transfected as indicated. 48 h post-transfection, cells were harvested and lysed. 5% of the lysate was used for IB to visualize HA-tagged receptors, (Flag Ub)n-proteins, and Etag KLHL12, respectively (left panels). The rest of the lysate was subjected to IP with anti-HA (16B12). After IP proteins were eluted in 0.5% SDS at 95°C. Next, lysates were incubated with or without addition of 50 mM NaOH for 1 h at 32°C, mock treated samples were incubated with PBS. After dilution with RIPA lysis buffer a second round of IP was performed with anti-HA antibody. Specific purification of the receptors after the second IP was confirmed upon IB with rabbit anti-HA (1:2000), whereas receptor ubiquitination was revealed upon IB with anti-Flag-HRP (right panel, 1:2000). N: samples treated with 50 mM NaOH.
(TIF)
