# Supplementary figures and images for: KLHL12 Promotes Non-Lysine Ubiquitination of the Dopamine Receptors D4.2 and D4.4, but Not of the ADHD-Associated D4.7 Variant
Source: PLoS One. 2015 Dec 30;10(12):e0145654. doi: 10.1371/journal.pone.0145654 (PMC4738440; doi:10.1371/journal.pone.0145654)

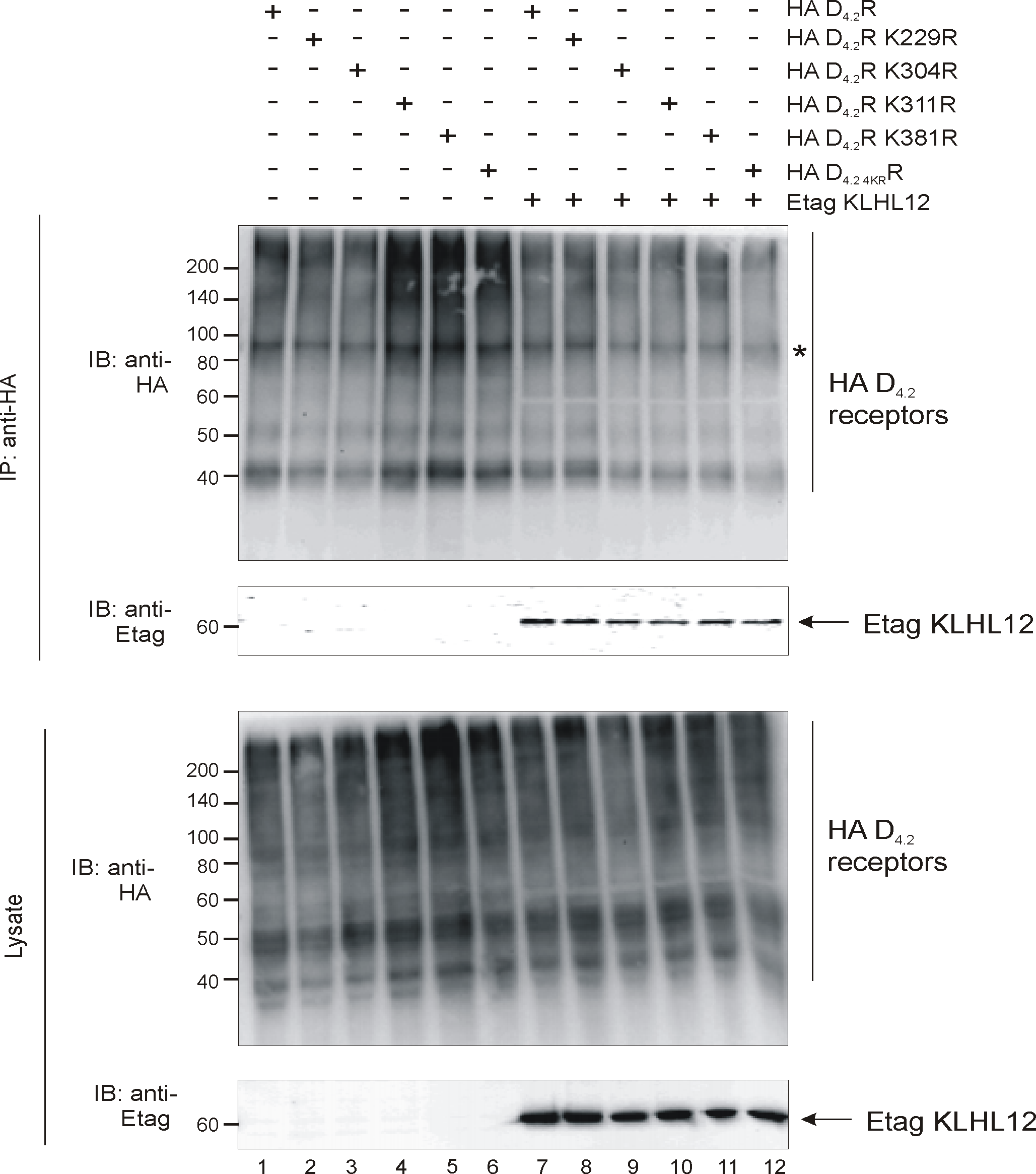

Supplement: S1 Fig — HEK293T cells were transiently transfected as indicated. 48 h post-transfection, cells were harvested and lysed. 5% of the lysate was used for IB to visualize HA-tagged D4 receptors and Etag KLHL12. The rest of the lysate was subjected to IP with anti-HA (16B12). Specific purification of the receptors after IP was confirmed upon IB with anti-HA (1:2000), whereas co-precipitation of Etag KLHL12 was verified by IB with anti-Etag (1:2000). * Association of two heavy chains of mouse anti-HA antibody (each 50 kDa). (TIF) [file pone.0145654.s001.tif]

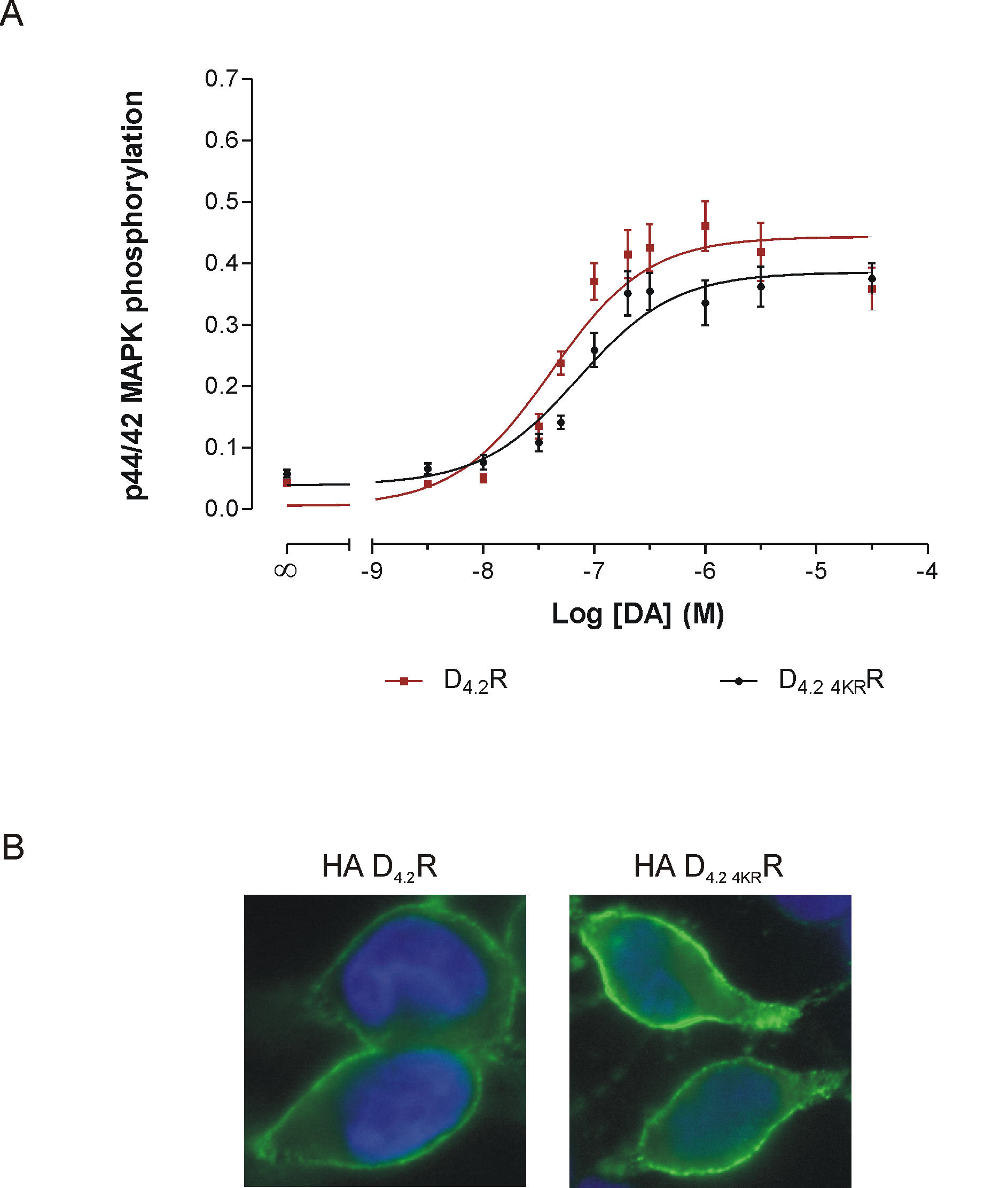

Supplement: S2 Fig — A) HEK293S cells stably expressing HA D4.2R and HA D4.2 4KRR were treated with dopamine in concentration ranging from 10−8.5 to 10−4.5 M. An in cell western assay was performed and phosphorylated p44/42 MAP kinase was detected with rabbit anti-phospho p44/42 MAPK (1:1000). Values were normalized against the signal detected with mouse anti-p44/42 MAPK (1:800). B) HEK293S cells stably expressing HA D4.2R and HA D4.2 4KRR were seeded in wells with coverslips. Cells were stained using primary antibody rabbit anti-HA (1:1000) and secondary antibody anti-rabbit Alexa Fluor 488 (1:500). Cell nuclei were colored with DAPI. (TIF) [file pone.0145654.s002.tif]

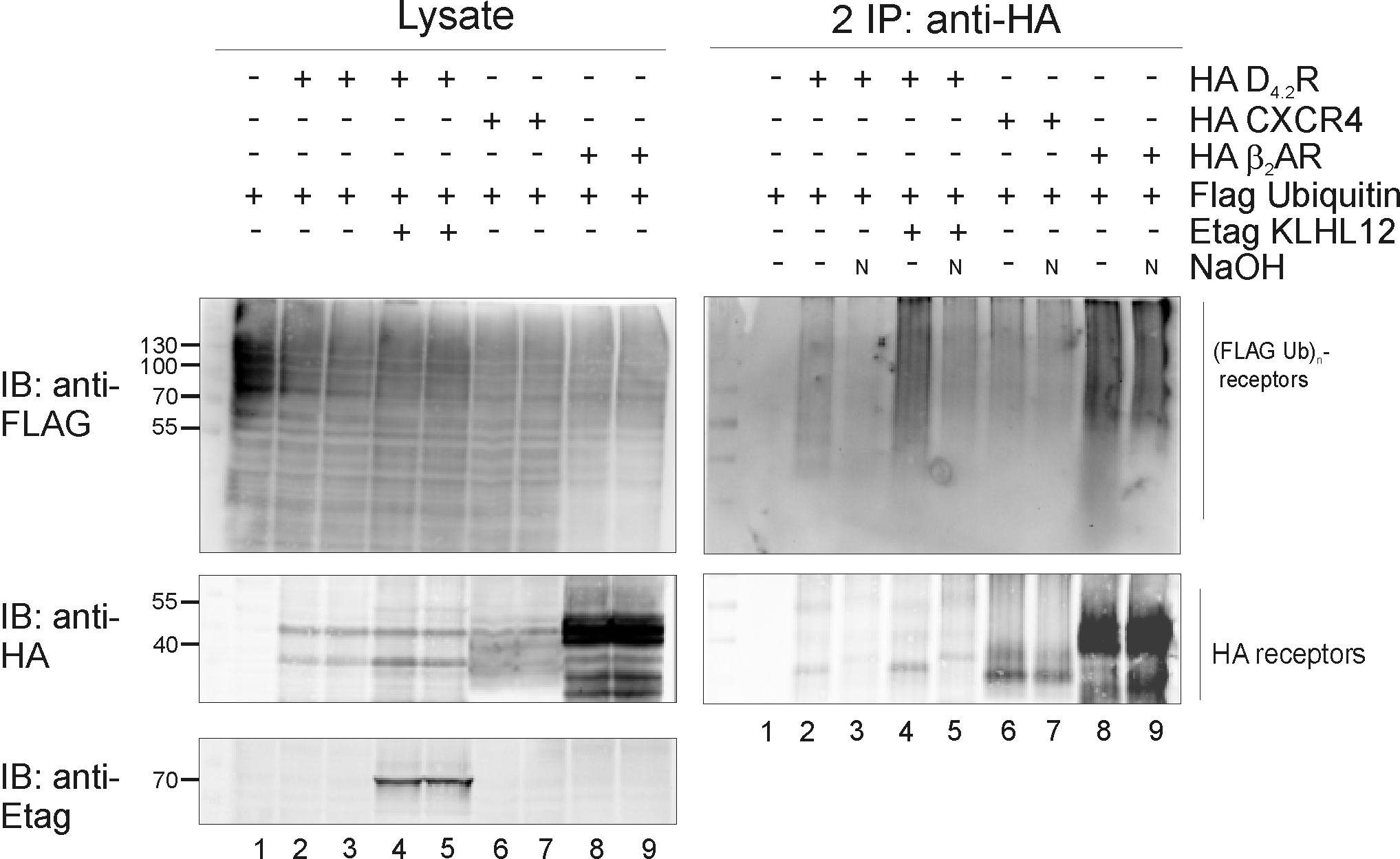

Supplement: S3 Fig — HEK293T cells were transiently transfected as indicated. 48 h post-transfection, cells were harvested and lysed. 5% of the lysate was used for IB to visualize HA-tagged receptors, (Flag Ub)n-proteins, and Etag KLHL12, respectively (left panels). The rest of the lysate was subjected to IP with anti-HA (16B12). After IP proteins were eluted in 0.5% SDS at 95°C. Next, lysates were incubated with or without addition of 50 mM NaOH for 1 h at 32°C, mock treated samples were incubated with PBS. After dilution with RIPA lysis buffer a second round of IP was performed with anti-HA antibody. Specific purification of the receptors after the second IP was confirmed upon IB with rabbit anti-HA (1:2000), whereas receptor ubiquitination was revealed upon IB with anti-Flag-HRP (right panel, 1:2000). N: samples treated with 50 mM NaOH. (TIF) [file pone.0145654.s003.tif]
